# Music therapy preferences among breast cancer patients undergoing perioperative chemotherapy: from qualitative insight to quantitative evidence

**DOI:** 10.1007/s10147-025-02872-5

**Published:** 2025-09-03

**Authors:** Nanami Nakaya, Ami Yamasato, Mayu Kondo, Shigeki Okino, Atsuko Kitano, Banri Tsuda, Makoto Tokuhara, Kenji Yamamoto

**Affiliations:** 1 Graduate School of Medicine, Tokai University, Isehara City, Kanagawa Japan; 2 Department of Psychiatry, Tokai University School of Medicine, Isehara City, Kanagawa Japan; 3 Department of Art, School Humanities & Culture, Tokai University, Hiratsuka City, Kanagawa Japan; 4 Department of Oncology, St. Luke’s International Hospital, Chuo-ku, Tokyo Japan; 5 Department of Palliative Medicine, Tokai University School of Medicine, Isehara City, Kanagawa Japan

**Keywords:** Music therapy, Palliative care, Preference, Request, Breast cancer

## Abstract

**Background:**

Patients with breast cancer (PWBC) have a high risk of developing psychological disorders, and interventions should be implemented with care. For music therapy (MT) interventions to be more effective among these patients, providing therapeutic programs tailored to their preferences is desirable. Given the limited research on the preferences for MT of PWBC undergoing chemotherapy, we had previously conducted a qualitative study to clarify their preferences, yielding the formation of related hypotheses. Building upon these hypotheses, the current quantitative study aimed to clarify the specific preferences regarding MT of PWBC, who are undergoing chemotherapy, in Japan.

**Methods:**

This quantitative study’s questionnaire was created based on the results of our prior qualitative study and completed by 300 PWBC undergoing perioperative chemotherapy.

**Results:**

(a) Of all participants, 80.9% wanted MT, and there was a relationship between preferred activity type and participants’ sociodemographic and clinical characteristics; (b) the expectations of participants regarding MT differed by preferred activity type; and (c) the participants’ preferences regarding the frequency, timing, duration, and cost of the MT intervention were constant regardless of the preferred activity type.

**Conclusions:**

As patients’ specific preferences have been clarified, we conclude that professionals may be more well-positioned and informed to devise more appropriate and effective MT programs to be incorporated into the treatment schedules of PWBC who are undergoing chemotherapy, with vital quality-of-life implications resulting.

## Introduction

The effectiveness of music therapy (MT) in patients with cancer has recently been reported, with meta-analyses and reviews conducted to clarify this effectiveness showing that such therapy and music-based interventions improve quality of life (QoL), anxiety, depression, and pain in patients with cancer [1–3]. These studies also illustrate that MT and music-based interventions for this population may be effective in various treatment situations (e.g., before and after tests and surgery, during chemotherapy, and during palliative care), regardless of cancer type. Furthermore, this therapy may be useful for alleviating physical problems (e.g., pain), low life quality, and psychological issues (e.g., depression and anxiety) caused by cancer and its treatment.

Breast cancer is the most common cancer among women, accounting for 11.6% of all cancers worldwide [4]. Patients with breast cancer (PWBC) are also at high risk of developing psychological disorders, e.g., depression and anxiety [5], with a global depression prevalence among these patients of 32.2% [6]. Some studies have reported that receptive MT significantly improves symptoms of pain, depression, fatigue, and sleep disorders [7–9]. However, these studies often developed the MT protocol based on the music therapist’s experience rather than on a standardized format, considering participants, techniques, or duration of therapy.

The consensus is that MT for patients with cancer should be tailored to the type of cancer afflicting the patient and their unique treatment situation (e.g., whether before or after tests and surgery or during chemotherapy and palliative care) [10]. However, limited research has examined patient preferences. One study reported that 88% of patients with cancer undergoing treatment for three months expressed positive opinions on MT [11]. Before the authors’ initial qualitative study [12], no studies in Japan had examined the preferences or opinions of patients with cancer regarding MT or the preferences of PWBC undergoing treatment or MT protocols.

To address the gap in patient-informed MT design, we conducted a qualitative study to clarify the preferences of Japanese PWBC regarding MT during chemotherapy [12]. The results were classified into three categories: relationship to music, pain from treatment, and preferences for MT. The analysis showed that patients undergoing chemotherapy determine their MT preferences based on their own musical experiences and physical and psychological pain. Furthermore, subcategories were created for patients’ expectations regarding the content, frequency, duration, and cost—among other factors—of MT. An overview of the MT expectations of PWBC undergoing perioperative chemotherapy was presented. Subsequently, to determine whether the content of the authors’ prior qualitative research represents the opinions of most PWBC undergoing perioperative chemotherapy, a larger-scale quantitative study was deemed necessary. Thus, building on prior qualitative findings, this study aimed to quantitatively examine MT preferences—across dimensions such as activity type, timing, frequency, duration, cost, and therapeutic expectations—among PWBC undergoing perioperative chemotherapy in Japan.

## Methods

### Participants

Participants were adults who (1) had been diagnosed with primary breast cancer and were undergoing or had undergone perioperative chemotherapy at University Hospital A or Hospital B, or (2) were involved in Breast Cancer Support Community C, had been diagnosed with primary breast cancer, and were undergoing or had undergone perioperative chemotherapy. All participants read the explanatory document and provided informed consent.

The doctors in charge at University Hospital A and Hospital B recruited participants by distributing flyers to viable candidates. These candidates accessed and answered the questionnaire online using the URL and QR code provided on the flyer. Breast Cancer Support Community C was similarly asked to include the questionnaire URL and QR code in a recruitment-focused email and distribute it to potential participants.

The questionnaire was developed using subcategories obtained from the prior qualitative study, with the corresponding codes used as multiple-choice options. It was validated by experts, including music therapists, psychiatrists, breast oncologists, and palliative care physicians. The application period spanned September 2021–November 2021.

### Data collection

The questionnaire was created based on the results of a qualitative study [12] conducted using Charmaz’s [13] constructivist grounded theory approach. The multiple-choice questionnaire collected data on respondents’ current age, age at diagnosis, marital status, educational level, occupation, current treatment status, and chemotherapy type, as well as their preferred MT format (group or IT), expectations, frequency, timing, duration, and cost. Data were collected online using Questant [14]—a self-administered questionnaire tool provided by Macromill, Inc.

### Analysis

Descriptive statistics were utilized to analyze the data collected using the questionnaire. Pearson’s chi-squared test and the Kruskal–Walis test were employed to investigate the relationship between participants’ sociodemographics, clinical characteristics, and preferred activity type, with *p* < 0.05 expressing statistical significance in both tests. A residual analysis was conducted for items with a significant difference in Pearson’s chi-squared test; for items with a significant difference in the Kruskal–Walis test (*p* < 0.05), the groups were compared. A Pearson’s chi-squared test (*p* < 0.05) was conducted to assess differences in expectations between group and IT when both were desired. Items with a significant difference underwent a residual analysis. SPSS version 26 was used for all statistical procedures.

To uphold the findings’ reliability and validity, the research team (comprising psychiatrists, music therapists, breast oncology specialists, and musicology experts) met once a week to review data collection methods, share data, and confirm analysis methods and results.

## Results

### Participants’ sociodemographic and clinical characteristics

The study included 300 participants aged 21–84 (*M* = 52.2 years, SD = 12.4), with an age at diagnosis of 20–77 (*M* = 47.7 years, SD = 11.9). Table 1 shows participants’ sociodemographic and clinical characteristics.

**Table 1:** Profile of the participants

Items	Mean
Age	52.2 (21–84)
Age of morbidity	47.7 (20–77)

Items	Factors	Number of People (%)
Marital Status	Married	214 (71.3%)
	Unmarried	61 (20.3%)
	Divorced	16 (5.3%)
	Widowed	9 (3.0%)
	Other	0 (0.0%)
Education	High school	146 (48.7%)
	University	70 (23.3%)
	Junior college	62 (20.7%)
	Graduate school	10 (3.3%)
	Junior high school	6 (2.0%)
	Other	6 (2.0%)
Work	Housewife	104 (35.7%)
	Full-time employee	98 (32.7%)
	Part-time employee	58 (19.3%)
	Without an occupation	32 (10.7%)
	Student	1 (0.3%)
	Other	7 (2.3%)
Progress of Chemotherapy	After treatment	180 (60.0%)
	During treatment	118 (39.3%)
	No response	2 (0.6%)
Chemotherapy	EC Protocol	144 (48.0%)
	Docetaxel	127 (42.3%)
	Paclitaxel	101 (33.7%)
	TC Protocol	31 (10.3%)
	AC Protocol	27 (9.0%)
	FEC Protocol	13 (4.3%)
	Capecitabine	12 (4.0%)
	DoseDence-AC Protocol	6 (2.0%)
	Other	46 (15.3%)
	No response	11 (3.7%)

EC = Epirubicin + Cyclophosphamide; AC = Doxorubicin + Cyclophosphamide; FEC = Fluorouracil + Epirubicin + Cyclophosphamide; TC = Docetaxel + Cyclophosphamide; Dose Dence-AC = Doxorubicin + Cyclophosphamide + Pegfilgrastim

### Factors associated with preferred activity type

Regarding preferred activity type, 93 participants (31.0%) chose “group therapy” (GT), 88 (29.3%) “individual therapy” (IT), 61 (20.3%) “I want to receive both,” and 57 (19.0%) “I don’t want to receive either.” In total, 80.9% of participants expressed interest in receiving some form of MT. Among those preferring neither (multiple responses were allowed), 26.3% answered “because it is physically painful,” meaning they are in too much pain to consider receiving therapy, and 22.8% responded “because it is not psychologically painful” and therefore do not need any pain-relieving therapies.

Analyses using participants’ current age revealed that people preferring GT were older than those preferring IT (95% CI, 2.6852–11.5480) and older than those preferring both (95% CI, 5.4864–15.3498). People preferring IT were younger than those preferring GT and younger than those preferring neither (95% CI, −14.243–−4.1288). People preferring both types of therapy were younger than those preferring only GT and younger than those preferring neither (95% CI, −17.9803–−6.9804). People preferring neither were older than those preferring IT and older than those preferring both types of therapy.

Analyses using age at diagnosis revealed that people preferring GT were older than those preferring IT (95% CI, 2.8742–11.4875) and older than those preferring both types of therapy (95% CI, 4.3789–13.9646). People preferring IT were younger than those preferring GT and younger than those preferring neither (95% CI, −13.7321–−3.9107). People preferring both were younger than those preferring GT and younger than those preferring neither (95% CI, −16.1545–−5.4701). People preferring neither type of therapy were older than those preferring IT and older than those preferring both types of therapy.

Those preferring GT had significantly higher rates of “being a high school graduate or lower” and “currently undergoing chemotherapy.” They also exhibited significantly lower rates in “university graduate or higher” and “after chemotherapy.” Participants preferring IT were significantly more likely to be “working full-time” and “after chemotherapy,” and significantly less likely to be a “high school graduate or lower” and “currently undergoing chemotherapy.” Participants preferring both treatments were significantly more likely to be “university graduate or higher” and “work full-time” and significantly less likely to be a “high school graduate or below.” Participants not wanting to receive either treatment were significantly more likely to be “unemployed or a student” and significantly less likely to “work full-time.” Table 2 shows further details.

**Table 2:** Influence of participants’ sociodemographic and clinical characteristics on preferred activity type

Variable	Responses	Group therapy	Individual therapy	Want to take both	Do not want to take either	*p*-value
Number of participants who prefer the activity type		93	88	61	57	
Current age		▲55.935	▼48.818	▼45.517	▲58.086	*p* < 0.001**
Age at diagnosis		▲51.272	▼44.091	▼42.1	▲53.069	*p* < 0.001**
Marital status	Married	66 (71.0%)	58 (65.9%)	44 (72.1%)	46 (79.3%)	n.s
	Unmarried	16 (17.2%)	24 (27.3%)	13 (21.3%)	8 (13.8%)	
	Divorced or widowed	11 (11.8%)	6 (6.8%)	4 (6.6%)	4 (6.9%)	
Educational level	High school or lower	▲56 (60.2%)	44 (50.0%)	▼17 (27.9%)	35 (60.3%)	*p* < 0.001*
	University or higher	▼34 (36.6%)	43 (48.9%)	▲42 (68.9%)	22 (37.9%)	
Work	Full-time employee	23 (24.7%)	▲37 (44.3%)	▲27 (44.3%)	▼10 (17.2%)	*p* < 0.05*
	Part-time employee	19 (20.4%)	17 (19.3%)	9 (14.8%)	13 (22.4%)	
	Homemaker	39 (41.9%)	24 (27.3%)	20 (32.8%)	21 (36.2%)	
	Unemployed or student	12 (12.9%)	6 (6.8%)	4 (6.6%)	▲11 (19.0%)	
Progress of chemotherapy	Currently undergoing	▲46 (49.5%)	▼25 (28.4%)	22 (36.1%)	24 (41.4%)	*p* < 0.05*
	Completed	▼46 (49.5%)	▲62 (70.5%)	39 (63.9%)	33 (56.9%)	

^*^Pearson’s chi-squared test was applied to items other than current age and age at diagnosis, and a residual analysis was applied to items for which a significant difference was found

^**^The Kruskal–Walis test was applied to current age and age at diagnosis, and a comparison of the groups was conducted because a significant difference was found

▲Items that were significantly higher as a result of Tukey’s test and compared the groups

▼Items that were significantly lower as a result of Tukey’s test and compared the groups

### Frequency of MT, timing, duration, and cost

Participants were asked about their preferences regarding frequency of MT, timing, duration, and cost. Table 3 outlines the most commonly selected items for each category.

**Table 3:** Preferred frequency, timing, duration, and cost

Items	Factors	Group therapy	Individual therapy	Both therapies
		Number of people (%)	Number of people (%)	Number of people (%)
Frequency	Once per course	51 (61.3%)	54 (61.4%)	37 (60.7%)
	Once per two courses	30 (32.3%)	24 (27.3%)	12 (19.7%)
	Twice or more per course	5 (5.4%)	5 (5.7%)	7 (11.5%)
	Once per three courses	2 (2.2%)	2 (2.3%)	3 (4.9%)
	Once per four courses	1 (1.1%)	0 (0.0%)	1 (1.6%)
	No response	4 (4.3%)	3 (3.4%)	1 (1.6%)
Timing	On the day of chemotherapy	63 (67.7%)	55 (62.5%)	26 (42.6%)
	Between 8 and 14 days after chemotherapy	14 (15.1%)	10 (11.4%)	8 (13.1%)
	Between 15 and 21 days after chemotherapy	9 (9.7%)	15 (17.0%)	16 (26.2%)
	22 days or more after chemotherapy	2 (2.2%)	3 (3.4%)	3 (4.9%)
	Within 7 days of the day after chemotherapy	1 (1.1%)	4 (4.5%)	7 (11.5%)
	Any time is fine	1 (1.1%)	1 (1.1%)	1 (1.6%)
	No answer	3 (3.2%)	0 (0.0%)	0 (0.0%)
Reasons for requesting this timing	Because I do not need to go to the hospital again	47 (50.5%)	41 (46.6%)	21 (34.4%)
	Because it is not physically painful	40 (43.0%)	42 (47.7%)	26 (42.6%)
	Because it is psychologically painful	8 (8.6%)	17 (19.3%)	9 (14.8%)
	Because it is physically painful	3 (3.2%)	5 (5.7%)	9 (14.8%)
	Because it is not psychologically painful	2 (2.2%)	7 (8.0%)	2 (3.3%)
	Because I think it will motivate me to get treatment	2 (2.2%)	12 (13.6%)	17 (27.9%)
	Other	4 (4.3%)	5 (5.7%)	1 (1.6%)
	No response	2 (2.2%)	0 (0.0%)	1 (1.6%)
Duration	Less than 30 min	41 (44.1%)	50 (56.8%)	21 (34.4%)
	31 to 60 min	41 (44.1%)	32 (36.4%)	37 (60.7%)
	61 to 120 min	6 (6.5%)	4 (4.5%)	1 (1.6%)
	More than 121 min	3 (3.2%)	2 (2.3%)	2 (3.3%)
	No response	2 (2.2%)	0 (0.0%)	0 (0.0%)
Cost	501–1000 yen	37 (39.8%)	28 (31.8%)	20 (32.8%)
	0–500 yen	29 (31.9%)	26 (29.5%)	14 (23.0%)
	1001–2000 yen	10 (10.8%)	12 (13.6%)	10 (16.4%)
	2001–3000 yen	10 (10.8%)	9 (10.2%)	11 (18.0%)
	3001–5000 yen	4 (4.3%)	5 (5.7%)	1 (1.6%)
	5001 yen or more	0 (0.0%)	2 (2.3%)	1 (1.6%)

#### Preferred frequency

Regardless of preferred activity type, “once per chemotherapy course” was the most common response, chosen by 61.3% of those preferring GT, 60.7% preferring IT, and 60.7% preferring both therapy types.

#### Preferred timing

Regardless of preferred activity type, the most common response was “on the day of chemotherapy,” chosen by 67.7% of those preferring GT, 62.5% preferring IT, and 64.2% preferring both therapy types. The reasons cited were “because I do not need to go to the hospital again,” “because it is not physically painful,” and “because it is psychologically painful.”

#### Preferred duration

The most common responses for those preferring GT were “less than 30 min” and “31–60 min,” with 44.1% of respondents choosing either option. The most common response (56.8%) for those preferring IT was “less than 30 min.” The most common response (60.7%) for those preferring both therapy types was “31–60 min.”

#### Cost

Regardless of preferred activity type, the most common response was “501–1000 yen,” chosen by 39.8% of those preferring GT, 31.8% preferring IT, and 32.8% preferring both therapy types, expecting to pay this amount (rate on July 22, 2025: 1 US dollar = 147.69 yen).

### Expectations regarding MT

Based on the author’s previous qualitative research [12], it was hypothesized that the current findings would differ depending on the participants’ preferred activity type. Accordingly, participants wanting to receive both types of therapy were asked to respond to questions regarding GT and IT separately, enabling examination of the differences between these two activity types (Table 4).

**Table 4:** Expectations for group and individual therapy

Items	Factors	Group therapy	Individual Therapy	Both Therapies		
				Group therapy	Individual Therapy	
		Number of people (%)	Number of people (%)	Number of people (%)	Number of people (%)	
Expectations	Interacting with others	▲73(78.5%)	▼17(19.3%)	▲46(75.4%)	▼24(39.3%)	*p* < 0.05
	Spending an enjoyable time	▲64(68.8%)	▼42(47.7%)	41(67.2%)	34(55.7%)	
	Feeling more energized	59(63.4%)	▼43(48.9%)	39(63.9%)	36(59.0%)	
	Relieving stress	▲55(59.1%)	39(44.3%)	31(50.8%)	25(41.0%)	
	Calming down	▼39(41.9%)	▲56(63.6%)	26(42.6%)	32(52.5%)	
	Increasing motivation for treatment and hospital visits	36(38.7%)	▼28(31.8%)	30(49.2%)	▲35(57.4%)	
	Alleviating physical side-effects	31(33.3%)	▲36(40.9%)	14(23.0%)	17(37.9%)	
	Improving singing or playing a musical instrument	14(15.1%)	5(5.7%)	7(11.5%)	7(11.5%)	
	Killing time	12(12.9%)	8(9.0%)	7(11.5%)	4(6.6%)	
	Other	0(0.0%)	1(1.1%)	0(0.0%)	0(0.0%)	
	No response	0(0.0%)	0(0.0%)	1(1.6%)	0(0.0%)	

Pearson's Chi-squared test was performed, and residual analysis was conducted for items that showed significant differences

▲Items that were significantly high as a result of residual analysis

▼Items that were significantly lower as a result of residual analysis

Those preferring GT expressed significantly higher expectations to “interact with others,” “have an enjoyable time,” and “relieve stress,” and significantly lower expectations to “calm the mind.” Those preferring IT expressed significantly higher expectations to “calm the mind” and “alleviate physical side effects” and significantly lower expectations to “interact with others,” “have an enjoyable time,” “feel more energized,” and “experience increased motivation for treatment and hospital visits.”

Those preferring both activity types exhibited significantly higher expectations to “interact with others” from GT. Regarding IT, they showed significantly higher expectations to “experience increased motivation for treatment and hospital visits” and significantly lower expectations to “interact with others” (*p* < 0.05).

## Discussion

This quantitative study used a questionnaire created based on the results of a previous qualitative study [12] to clarify the specific MT preferences of PWBC undergoing chemotherapy in Japan. First, this study identified high demand for MT among these patients (80.9% of participants expressing interest) and some variation in their preferred activity type (GT, IT, or both types). Second, regarding therapy that would best meet the needs of patients undergoing chemotherapy, most participants preferred MT once per chemotherapy course or once every two courses, scheduled on the day of treatment, regardless of their preferred activity type. The preferred duration was less than 30 min or 31–60 min, and most were willing to pay 0–500 or 501–1,000 yen for the therapy. Third, participants’ expectations regarding MT differed by preferred activity type, with those preferring GT expecting to “interact with others” and “feel energized,” and those preferring IT expecting to “calm the mind” and “alleviate physical side effects.”

### Preferences for MT among PWBC undergoing or previously having undergone perioperative chemotherapy

This study found that 31.3% of participants (out of 300) preferred GT, 29.3% IT, and 20.3% both, with 80.9% indicating they would like to receive some form of MT. Comparisons with prior research are challenging due to sample heterogeneity, which focused on the need for MT. However, a USA study found that 85% of 65 patients with cancer expressed an interest in music interventions [15]—a finding similar to that reported in the current study. Moreover, a study in Germany reported that 40% of 486 patients with cancer (across various types and treatments) expressed an interest in MT [16]. Similarly, another study indicated that approximately 30% of patients with terminal cancer in hospice settings expressed interest in MT [17].

Gotay and Lau [18] surveyed patients with cancer from various ethnic groups regarding their experiences receiving psychological support and their level of interest in it. Their interest varied by country, and no apparent differences in preferences existed. Another study noted that health beliefs, cultural background, and healthcare experiences varied by ethnicity among patients with cancer, making it difficult to meet diverse preferences and causing frustration [19]. This highlights the need for further research on the preferences of patients with cancer from different contexts regarding MT. It also reinforces the importance of cultural sensitivity in caring for patients with cancer globally [20].

In addition, studies have shown that younger patients experience more distress from cancer than older patients [15]. In the present study, those preferring IT and both therapy types tended to be significantly younger, both in current age and age at diagnosis. Notably, many participants who preferred only IT expected it to “calm the mind” and “alleviate physical side effects.” These findings suggest that patients who only preferred IT may have been experiencing strong psychological distress—consistent with prior research [15]. The past reviews have emphasized that young patients with cancer experience more intense physical and psychological pain, highlighting the need for care responsive to their specific needs. These reviews have also raised concerns regarding clinicians being insufficiently sensitive to the unique preferences and values of the younger generation [21]. To better meet these needs, we recommend reaching out for individualized support tailored to young patients’ circumstances.

### MT intervention’s frequency, timing, duration, and cost

To the authors’ knowledge, this is the first quantitative investigation aimed at clarifying specific preferences regarding MT interventions’ frequency, timing, duration, and cost among PWBC who prefer either GT or IT. The findings suggest that offering MT once every one to two chemotherapy courses on the treatment day for under 60 min and at less than 1,000 yen (= 6.77 US dollars) may meet many patients’ preferences, regardless of activity type.

A notable study reported that the influence of MT frequency on its effectiveness is unknown [1], although another study stated that a duration of “more than one month and less than two months” is most effective for improving the QoL of patients with cancer [3]. However, the literature also indicates that professionals face difficulties in designing an MT protocol that can be reasonably incorporated into different treatment schedules; some patients with cancer struggle to visit hospitals for MT, although others are reluctant to discuss their psychological distress with medical staff [10, 22]. Nevertheless, considering the specific patient preferences identified in this study, responding flexibly to patient needs may make it feasible to incorporate more systematic MT into treatment schedules.

### Expectations regarding MT

Among those preferring GT and both therapy types, many participants expected to “interact with others”—consistent with previous qualitative research [12] and studies noting the “psychological effects of interaction and sharing of emotions between patients” in GT [23]. In GT for patients with cancer, sharing emotions with others who have the same illness helps them feel less unhappy and isolated although reassuring them their reactions are normal [24].

In this study sample, those preferring GT were significantly more likely than those preferring IT to view MT as an active and positive intervention, expecting to “have an enjoyable time” and “relieve stress.” Those preferring IT had significantly higher expectations to “calm the mind” and “alleviate physical side effects,” seeking relief from their psychological distress. These results may be due to the younger age of those preferring IT, who were more likely to experience psychological distress [15]. According to qualitative research, patients determine their preferences based on their relationship with music. Many patients enjoy singing, but those not good at singing dislike it. Patients with musical instrument experience prefer musical activities, and those with songwriting experience enjoy songwriting [12]. Various activities’ effectiveness has been studied, and by considering these results and tailoring activities to individual patients, it is possible to meet many patients’ preferences [25, 26]. The studies highlight the importance of using MT to alleviate pain in patients with cancer, as it helps reduce psychological and physical problems [7, 9, 25–31]. These findings may help music therapists develop specific protocols for applying MT interventions to PWBC undergoing chemotherapy.

### Limitations

This study has certain limitations. All participants were female PWBC undergoing chemotherapy, and so caution is needed when generalizing the findings to male patients, patients with other cancer types, or those undergoing other treatment types. Moreover, the lack of samples from other cultures, countries, and healthcare systems also limits the findings’ applicability beyond the present study’s context.

The profile of the participants and their expectations of MT differed according to their preferred type of activity. However, this study does not prove a causal relationship, and additional research is needed to examine these associations.

Perioperative chemotherapy may have different effects on physical and psychological pain depending on the treatment regimen, and differences in treatment regimens may have influenced the results of this study. Owing to the wide variety of treatment regimens used in this study, it was not possible to clarify the differences, and so further investigation is needed.

## Conclusion

This study explored the preferences of PWBC undergoing chemotherapy for MT, specifically regarding their expectations, timing, frequency, duration, cost, and activity types. The results indicate a high need and preference for MT among patients undergoing preoperative and postoperative chemotherapy in Japan, offering important insights for planning patient-centered care. The findings can assist healthcare professionals in selecting MT types that align with treatment schedules and patient needs in a clinical context in Japan. Furthermore, careful consideration is needed to ensure the therapy type aligns with patients’ expectations and effectively addresses their symptoms. This study’s results also support and clarify the previous qualitative studies’ hypotheses. Notably, despite the inclusion of an “other” category in the questionnaire—designed based on earlier qualitative findings—most participants selected predefined options, reinforcing the prior coding framework’s validity. Looking ahead, further research is needed to evaluate the effectiveness of interventions developed based on these preferences, examine how this preference framework applies to other populations, and explore the feasibility of implementing such interventions in clinical practice.

## Data Availability

The data that support the findings of this study are not openly available due to reasons of sensitivity and are available from the corresponding author upon reasonable request.
